# Characteristics of sudanese camel-hair fibres under subtropical desert condition

**DOI:** 10.1007/s11250-024-03951-x

**Published:** 2024-03-25

**Authors:** Rania Agamy, Sabry F. Mahouda, Ali H. Azzam, Aymen A.I. Gad Allah, Ibrahim I. Abdel-Mageed

**Affiliations:** 1 Animal Production Department, Faculty of Agriculture, Cairo University, Giza, Egypt; 2 Animal and Poultry Division, Desert Research Center, Cairo, Egypt

**Keywords:** Amino acids, Fibre quality, Hair fibre diameter, Minerals, Sudanese camel

## Abstract

The study assessed the quality and variability of camel hair fibres in arid regions of Egypt. Raw camel-hair samples were collected from fifteen Sudanese camels divided into seven males (414.60 ± 38.19 kg, BW) and eight females (401.67 ± 26.76 kg BW), and the study investigated the influences of animal sex on both the physical and chemical traits of camel-hair fibers. The relationships among physical properties and both mineral and amino acid content were studied. Camel’s sex had no significant effect on any of the studied traits including fibre diameter (FD), prickle factor (PF), medullated fibre (MF), staple length (SL) and staple strength (SS). In the meantime, no significant differences were found between males and females in fibers’ minerals contents except potassium, where fibres of females had significantly higher potassium content than those of males. For amino acids contents in camel fibres, camel sex had a significant effect only on glutamic acid, since fibres of males showed higher (*P* < 0.05) content than females. Fibre diameter had positive (*P* < 0.01) correlations with prickle factor (*r* = 0.83) and medullated fibres (*r* = 0.73). Zinc content in camel fibres was positively correlated with fibre diameter (*r* = 0.57; *P* < 0.05) and medullated fibres (*r* = 0.73; *P* < 0.01). Moreover, a significant (negative correlation coefficient *P* < 0.05) was found between fibre diameter and both sulfur and proline contents (*r*=-0.39 and − 0.56). Ammonia content in fibres was correlated negatively (*P* < 0.05) with prickle factor and elongation (*r*=-0.62 and − 0.58, respectively). The variability in the physical properties and chemical composition of Sudanese camel-hair fibers under subtropical desert conditions may shed light on the possibility of improving fiber quality.

## Introduction

Sudanese camels belong to the dromedary camels (single-humped) found throughout Africa, Arab countries, and Near Eastern and, in a lower extent, in southern regions of Asia. They may sustain life, perform well and produce high quality products such as meat, milk and fibres under harsh desert conditions (Wu et al., 2014). According to FAO STATS, the population of camels around the world is about 40 million; nearly 34 million one-humped dromedaries are raised in dry Africa areas, mainly in the North East (FAO, 2021). Camels have long been bred and raised for their meat, milk, manure, skins, and fibers, in addition to being used for transportation (Sahani et al., 2006).

Camel’s hair is classified as a specialty hair fibre. The specialty fibres are costly due to their luxurious texture and scarcity. Specialty fibres are rare animal fibres, such as mohair, cashmere, alpaca, and camel hair which have unique properties including luster, softness, natural color, and give warmth. These fibres represent a small portion of global fibre resource; specialty fibers become most valuable when combined in optimal proportions with other types of fibers. They enhance and alter the texture and appearance of the end products, whether used alone or in blended forms (Anjali and Suman, 2013).

The identification, classification and quantitative determination of animal fibers are a major challenge mainly for textile fraud control, fashion, forensics and archeological fields (Zoccola et al., 2023). Animal fibers are made up of eighteen amino acids and characterized by the abundance of the amino acids’ cysteine, which forms sulfur intra and inter-molecular chain bonds that confer the protein named keratin, a high chemical resistance (Maclaren and Milligan, 1981).

In Egypt, camels population (*Camelus dromedarius*) is about 120,000 heads that produce around 144 tons of hair yearly (FAO, 2021); nevertheless, only a little quantity used by Bedouin tribes to create traditional clothes. Basic information on camel’s hair characteristics in Egypt is limited. Therefore, analyzing the physical and chemical characteristics of Sudanese camel fibers according to the sex of animals can provide valuable insights and contribute to several areas of research and application. Understanding the properties of camel fibers according to sex can inform product development strategies, catering to specific market demands for textiles, rugs, or other camel fiber-based products. Moreover conducting a correlation study between the physical characteristics and chemical composition of camel fibers is essential for optimizing breeding programs, guiding product development in industries, and enhancing overall fiber quality and sustainability. This research aimed to analyze the physical and chemical properties of Sudanese camel fibers based on the animals’ sex.

The correlations between the physical and chemical properties of Sudanese camel fibers were also tested.

## Materials and methods

### Experimental animals and sampling

The experiment was conducted at Ras-Sudr Research Station, South Sinai Governorate, Desert Research Center, about 200 km from Cairo, Egypt. This study was conducted under guidelines set by the Animal and Poultry Production Division, Desert Research Center, Egypt, where the average temperature was 27.5^◦^C and the humidity was 53%. Fifteen adult Sudanese camels (*Camelus dromedaries*) of both sexes (seven males (414.60 ± 38.19 kg, BW) and eight females (401.67 ± 26.76 kg BW)) were used to collect hair fibre samples during the shearing season in spring 2020. Animals were randomly selected from the same healthy flocks that are subjected to same management system. Males and females were kept separately in shaded yards (20 × 20 m^2^). All animals fed on the same diet, which consisted of *ad libitum* of alfalfa hay and concentrates feed mixture, 2% as DM of their body weight according the recommendation of Askar et al., (2023) with free access to drinking water. Concentrate feed mixture consisted of 15% wheat bran, 55% yellow corn, 15% soybean meal, 10% cottonseed meal, 1.5% common salt, 2.5% limestone, 0.5% sodium bicarbonate, 0.3% premix, 0.1% antitoxins, and 0.1% yeast. Camels’ hair samples (50 gm.) representing each animal were cut with scissors at skin level from the right mid-side of the animal, close to the ribs. The proximate chemical composition of the alfalfa hay and concentrates feed mixture is shown in Table 1.

**Table 1:** The chemical composition of concentrate feed mixture and alfalfa hay

Nutrients	Concentrate feed mixture	Alfalfa hay
Dry matter (DM), %	94.6	93.8
Organic matter (OM), %	87.4	80.9
Crude protein (CP), %	15.6	14.1
Neutral detergent fiber (NDF), %	34.2	46.4

### Physical properties of camels’ fiber

Hair samples were analyzed at the Central Laboratory of Desert Research Center, Cairo, Egypt. Objective measurements and chemical composition; minerals, and amino acids contents were measured for raw hair fibres. Twenty staples were taken randomly to represent each animal after sheering. These staples were used to measure staple length (SL), according to Booth (1964). Using a LEICA Q 500 MC (User manual Q-500-MC image processing and analysis system manual) and a 4/0.12 lens, the fibre diameter (FD) was determined in microns. About five hundred fibres chosen at random were measured from each staple. It was ensured that each fibre was measured only once to avoid any duplication. The mean fibre diameters were calculated for each sample. The numbers of medullated fibres (MF) were counted and their percentages were calculated and recorded for all samples. Prickle factor (PF) was calculated from the distribution of fibre diameter in each sample as the proportion of fibres with diameters greater than 30 μm from the total number of fibres According to Helal et al. (2019). Twenty staples for each animal were used to measure strength using Agritest Staple Breaker (Caffin, 1980). Staple strength (SS) indicates the force required to break down the staple and divide that value by staple thickness (Newton per kilotex). The length of the top and the base of each broken staple in the strength test were measured and then collected. The increase in length as a percentage of the initial staple length was used to calculate the elongation (EL) percentage, as reported by El-Gabbas et al. (1999).

### Chemical properties of camels’ fiber

Camel-hair mineral contents (aluminum, barium, cadmium, cobalt, chromite, copper, iron, manganese, molybdenum, nickel, lead, silicon, strontium, vanadium, zinc, calcium, barium, potassium, sodium, and sulfur) were performed using Inductively Coupled Argon Plasma, ICAP, 6500 Duo, Thermo Scientific, England. One thousand mg/L multi-element certified standard solution, Merck, Germany, was used as a stock solution for instrument standardization and Flame Photometer (PFP7, Jenway, UK instrument) by the procedure of ASTM (2002).

The Amino acid analyzer (Sykam S4300 Amino Acid Reaction Module SYKAM GmbH, Munich, Germany) was used to determine the amino acid content of camel-hair after hydrolysis of samples according to the methodology of Pellet and Young (1980). The amino acids that were measured are aspartic acid, threonine, serine, glutamic acid, glycine, alanine, cysteine, valine, methionine, isoleucine, leucine, tyrosine, phenylalanine, histidine, lysine, arginine, and proline.

### Correlation coefficients

Correlation coefficients were calculated between each hair’s physical characteristics (prickle factor, medullated fiber, staple length, staple strength, and elongation), as well as between the hair’s physical characteristics and their mineral contents. Additionally, correlation coefficients were determined between the hair’s physical characteristics and their amino acids and ammonia contents.

### Statistical analysis

General linear model procedures of the SAS software (v. 9.3, SAS Inst. In., Cary, NC, USA, 2011) were used to analyze data. The main effects were animal sex (X_F_: Female and X_M_: Male). The used statistical model is.

Y_jk_ = µ + S_j_+ e_jk_.

Where Y_jk_: the k^th^ observation of the j^th^ sex. µ: the overall mean. S_j_: the effect of the j^th^ camel sex. e_jk_: Random error. All data are reported as least square means (LSM) ± standard errors (SE). Sex effect was assumed as a fixed except the random error, which was assumed independent and normally distributed with mean 0 and variance σ^2^. Each animal in the statistical model was considered as the experimental unit and the significance degree was detected at *P* ≤ 0.05. Using SAS (2011) comparisons among means within each classification were tested using Duncan’s Multiple Range Test. Simple correlation coefficients among various studied traits were calculated and tested for significance as mentioned before.

## Results

### Physical characteristics of camel-hair

Table (2) showed that the camel’s sex had no significant effect on fibre diameter (FD), prickles factor (PF), medullated fibre (MF), staple length (SL) and staple strength (SS). The coefficient of variation (CV %) reflecting variability for prickle factor and medullated fibre were high and valued at 52.98% and 50.96%, respectively.

**Table 2:** Physical characteristics of Sudanese camel-hair as affected of sex

Subclass description	N	FD (µm)	PF (%)	MF (%)	SL (cm)	SS (Ktex)	EL%	
Sex								
** X**_**M**_	7	34.5 ± 3.1	47.4 ± 13.4	33.6 ± 8.1	11.9 ± 1.4	44.9 ± 1.4	12.6 ± 0.78	
** X**_**F**_	8	32 ± 2.2	45.3 ± 9.4	27 ± 5.7	13.4 ± 0.96	40.8 ± 1.97	13.7 ± 1.11	
**P value**		0.57	0.90	0.58	0.43	0.16	0.51	
**Overall mean**	15	32.2 ± 5.37	44.3 ± 23.5	27.9 ± 14.2	12.8 ± 2.40	43.1 ± 3.44	13.1 ± 1.94	
** CV%**		16.7	52.9	50.9	18.7	7.97	14.9	
** R** ^**2**^		0.35	0.37	0.31	0.05	0.21	0.30	

Sub-class means with different superscripts differ significantly (*P* < 0.05). Least-square means (LSM), Standard error (SE), Number of animals (N), Fibre diameter (FD), Prickle factor (PF), Medullated fibre (MF), Staple length (SL), Staple strength (SS), Elongation (EL), Coefficient of variation (CV%), and Coefficient of determination (R^2^). sex of animal (X_M_: male and X_F_: female)

### Chemical characteristics of camel-hair

#### Minerals contents

Results presented in Table 3 indicate a comparative analysis of the trace element contents in both male and female hair samples. No significant differences were observed between the two studied groups in the content of trace elements, except for potassium hair sample of females had significantly (*P* = 0.0101) higher potassium content than males.

**Table 3:** Minerals content (ppm) of Sudanese camel-hair as affected of sex

Subclass description	Sex		*P* value	Overall mean	CV%	R^2^
	X_M_	X_F_				
**Aluminum (Al)**	515 ± 38.3	502 ± 26.9	0.82	507 ± 66.9	13.2	0.07
**Boron (B)**	2.60 ± 0.24	3.27 ± 0.17	0.07	3.05 ± 0.42	13.9	0.36
**Cadmium (Cd)**	0.05 ± 0.03	0.09 ± 0.02	0.46	0.07 ± 0.05	70.5	0.07
**Cobalt (Co)**	0.54 ± 0.06	0.53 ± 0.04	0.92	0.53 ± 0.10	18.4	0.01
**Chromite (Cr)**	36.1 ± 1.83	32.7 ± 1.28	0.21	34.2 ± 3.20	9.35	0.18
**Copper (Cu)**	22.5 ± 2.89	17.3 ± 2.02	0.22	19.8 ± 5.05	25.5	0.28
**Iron (Fe)**	526 ± 11.62	532 ± 8.14	0.69	529 ± 20.3	3.84	0.03
**Manganese (Mn)**	22.8 ± 1.60	24.1 ± 1.12	0.59	23.6 ± 2.80	11.9	0.03
**Molybdenum (Mo)**	2.01 ± 0.30	2.38 ± 0.21	0.39	2.26 ± 0.52	22.9	0.11
**Nickel (Ni)**	15.9 ± 1.41	16.3 ± 0.99	0.84	16.2 ± 2.46	15.2	0.01
**Lead (Pb)**	2.18 ± 0.28	2.16 ± 0.19	0.96	2.15 ± 0.48	22.5	0.07
**Silicon (Si)**	141 ± 13.22	157 ± 9.26	0.42	150 ± 23.11	15.4	0.07
**Strontium (Sr)**	39.9 ± 1.91	39.5 ± 1.34	0.87	39.6 ± 3.34	8.4	0.02
**Vanadium (V)**	1.06 ± 0.13	0.78 ± 0.09	0.16	0.88 ± 0.23	26.6	0.20
**Zinc (Zn)**	144 ± 6.81	132 ± 4.77	0.21	135 ± 11.90	8.81	0.41
**Calcium (Ca)**	4141 ± 479	4429 ± 336	0.68	4327 ± 837.57	19.4	0.02
**Barium (Ba)**	1.43 ± 0.28	1.63 ± 0.19	0.62	1.54 ± 0.49	31.6	0.04
**Potassium (K)**	1521 ± 182^b^	2299 ± 128^a^	0.01	1974 ± 318.07	16.1	0.48
**Sodium (Na)**	488 ± 36.8	525 ± 25.8	0.49	517.1 ± 64.3	12.4	0.24
**Sulfur (S)**	2586 ± 344	3290 ± 241	0.17	3006 ± 601	20	0.16

Sub-class means with different superscripts differ significantly (*P* < 0.05). Least-square means (LSM), Standard error (SE), Number of animals (N), Coefficient of variation (CV %), and Coefficient of determination (R^2^). sex of animal (X_M_: male and X_F_: female)

### Amino acids and ammonia contents

The results of the amino acid analyses of camel-hair fibres content are shown in Table (4). Camel hair of males and females contain considerable amounts of glutamic acid, arginine, serine and cysteine. Nevertheless, sex revealed a significant (*P* = 0.0285) effect in only glutamic acid since males’ fibres had a higher amount of glutamic acid than females.

Wide variations existed between the individual’s hair values for the arginine, tyrosine, glycine, aspartic acid and ammonia (114.22, 36.69, 55.17, 69.68 and 34.85 respectively in males and 107.79, 36.04, 52.26, 81.77 and 35.63 respectively in females). The CV% values were 10.36, 11.59, 18.85, 19.97 and 8.04% respectively (Table 4). However, the coefficient of variation for hair sample values of other amino acids varied within narrow limits (2.69, and 9.42%).

**Table 4:** Amino acids and ammonia contents (mg/gm) of Sudanese camel-hair as affected of sex

Subclass description	Sex		*P* value	Overall mean	CV%	R^2^
	X_M_	X_F_				
**Aspartic acid**	69.7 ± 8.90	81.8 ± 6.23	0.35	77.9 ± 15.5	19.9	0.14
**Threonine**	54.9 ± 1.99	56.6 ± 1.40	0.55	55.9 ± 3.49	6.25	0.03
**Serine**	106 ± 1.70	103 ± 1.19	0.32	104 ± 2.97	2.84	0.09
**Glutamic acid**	194 ± 7.49a	168 ± 5.24b	0.03	177 ± 13.1	7.37	0.38
**Glycine**	55.2 ± 5.76	52.3 ± 4.03	0.72	53.4 ± 10.1	18.9	0.01
**Alanine**	43.1 ± 1.56	45.6 ± 1.09	0.26	44.7 ± 2.72	6.09	0.13
**Cysteine**	107 ± 2.59	105 ± 1.81	0.59	106 ± 3.52	4.27	0.07
**Valine**	52.1 ± 2.35	52.6 ± 1.64	0.87	52.5 ± 4.10	7.82	0.01
**Methionine**	5.44 ± 0.24	4.93 ± 0.17	0.16	5.08 ± 0.42	8.30	0.38
**Isoleucine**	37.6 ± 1.90	34.6 ± 1.33	0.29	35.4 ± 3.33	9.42	0.36
**Leucine**	87.6 ± 1.35	88 ± 0.95	0.86	87.9 ± 2.37	2.69	0.01
**Tyrosine**	36.7 ± 2.40	36 ± 1.68	0.85	36.1 ± 4.19	11.6	0.13
**Phenylalanine**	38.5 ± 1.69	39.2 ± 1.19	0.77	39.1 ± 2.96	7.56	0.11
**Histidine**	15.3 ± 0.72	15.4 ± 0.50	0.90	15.3 ± 1.26	8.22	0.01
**Lysine**	35 ± 1.88	35.3 ± 1.31	0.92	35.2 ± 3.28	9.33	0.01
**Arginine**	114 ± 6.53	108 ± 4.58	0.50	110 ± 11.4	10.4	0.04
**Proline**	57.1 ± 2.57	64.4 ± 1.80	0.06	61.9 ± 4.49	7.25	0.31
**Ammonia**	34.85 ± 1.63	35.63 ± 1.14	0.7373	35.52 ± 2.85	8.04	0.13

Sub-class means with different superscripts differ significantly (*P* < 0.05). Least-square means (LSM), Standard error (SE), Number of animals (N), Coefficient of variation (CV %), and Coefficient of determination (R^2^). sex of animal (X_M_: male and X_F_: female)

**Table 5:** Correlation coefficients between physical characteristics of Sudanese camel-hair fibres

Physical traits	PF (%)	MF (%)	SL (cm)	SS (Ktex)	EL (%)
**FD (µm)**	0.83**	0.73**	0.02	0.04	0.47
**PF (%)**		0.35	0.22	0.18	0.53*
**MF (%)**			-0.04	-0.01	0.25
**SL (cm)**				0.51*	-0.03
**SS (Ktex)**					0.40

* = significant at *P* < 0.05; ** = significant at *P* < 0.01. Fibre diameter (FD), Prickle factor (PF), medullated fibre (MF), Staple length (SL), Staple strength (SS), Elongation (EL).

**Table 6:** Correlation coefficients between physical characteristics and minerals content (ppm) of Sudanese camel-hair fibres

Traits	FD(µm)	PF (%)	MF (%)	SL (cm)	SS (Ktex)	EL (%)
**Aluminum (Al)**	0.01	-0.1	0.26	-0.18	0.12	0.16
**Boron (B)**	-0.33	-0.26	-0.33	0.54*	0.27	-0.25
**Cadmium (Cd)**	-0.02	-0.06	0.04	-0.1	-0.37	-0.18
**Cobalt (Co)**	-0.12	-0.33	0.14	-0.28	-0.56*	-0.18
**Chromite (Cr)**	-0.1	-0.11	-0.09	-0.28	-0.58*	-0.05
**Copper (Cu)**	-0.22	-0.17	-0.22	-0.51*	-0.26	0.16
**Iron (Fe)**	-0.04	0.04	-0.05	0.64*	0.25	-0.26
**Manganese (Mn)**	0.33	0.18	0.27	-0.27	0.15	0.31
**Molybdenum (Mo)**	0.2	0.35	0.08	0.23	0.39	0.18
**Nickel (Ni)**	0.24	0.44	-0.17	-0.07	-0.27	0.55*
**Lead (Pb)**	0.19	0.19	-0.1	0.27	0.46	0.06
**Silicon (Si)**	0.16	-0.08	0.18	-0.27	0.08	0.26
**Strontium (Sr)**	-0.02	0.1	-0.18	-0.42	-0.45	0.19
**Vanadium (V)**	0.13	-0.11	0.07	-0.2	-0.33	0.02
**Zinc (Zn)**	0.57*	0.38	0.73**	-0.12	-0.05	0.27
**Calcium (Ca)**	-0.07	-0.3	0.28	-0.18	-0.27	0.03
**Barium (Ba)**	-0.08	-0.43	0.43	0.16	0.04	-0.37
**Potassium (K)**	0.2	0.2	0.17	0.26	0.62	0.38
**Sodium (Na)**	-0.13	-0.19	0.18	0.07	0.04	0.1
**Sulfur (S)**	-0.39*	-0.3	-0.16	-0.21	0.34	0.25

* = significant at *P* < 0.05; ** = significant at *P* < 0.01. Fibre diameter (FD), Prickle factor (PF), medullated fibre (MF), Staple length (SL), Staple strength (SS), Elongation (EL).

### Association between physical characteristics and chemical composition of camel-hair fibres

#### Correlation coefficients between physical characteristics

Fibre diameter was positively correlated (*P* < 0.01) with both prickle factor (*r* = 0.83) and medullated fibre percentage (*r* = 0.73), as shown in Table (5). The present study also shows positive correlations (*P* < 0.05) between elongation and prickle factor (*r* = 0.53). Longer staples were found to be significantly (*P* < 0.05) associated with higher staple strength (*r* = 0.51).

#### Correlation coefficients between physical characteristics, and minerals contents

Table (6) shows that zinc content of camel fibres was positively correlated with fibre diameter (*r* = 0.57, *P* < 0.05) and medullated fibre (*r* = 0.73, *P* < 0.01). Moreover, a significant (*P* < 0.05) negative correlation coefficient was found between fibre diameter and sulfur (*r*=-0.39). While staple length had a significant (*P* < 0.05) and positive correlation coefficients with both boron and iron (*r* = 0.54 and 0.64, respectively) and a negative correlation coefficient with copper (*r*=-0.51; *P* < 0.05). Contrarily, staple strength had a negative (*P* < 0.05) correlation coefficient with both cobalt (*r*= -0.56) and chromite (*r*=-0.58). The elongation had a positive and significant (*P* < 0.05) correlation coefficient with nickel (*r* = 0.55).

#### Correlation coefficients between physical characteristics, and amino acids and ammonia contents

Table 7 illustrates that fiber diameter had a significant (*P* < 0.05) negative correlation with proline (*r* = -0.56), medullated fiber was negatively correlated with both valine (*r* = -0.52, *P* < 0.05) and proline (*r* = -0.65, *P* < 0.01), while medullated fiber was positively correlated with isoleucine content in fibers (*r* = 0.57, *P* < 0.05); staple length showed a positive correlation coefficient (*P* < 0.05) with valine (*r* = 0.55), staple strength was negatively correlated with glutamic acid (*r* = -0.58, *P* < 0.05), and ammonia content in fibers was negatively correlated (*P* < 0.05) with both prickle factor and elongation (*r* = -0.62 and − 0.58, respectively).

**Table 7:** Correlation coefficients among physical characteristics and amino acids and ammonia content (mg/gm) of Sudanese camel-hair fibres

Traits	FD(µm)	PF (%)	MF (%)	SL (cm)	SS (Ktex)	EL (%)
**Aspartic acid (Asp)**	-0.20	-0.06	0.05	0.32	-0.06	-0.11
**Threonine (Thr)**	0.06	-0.26	0.32	-0.14	0.06	0.06
**Serine (Ser)**	-0.37	-0.09	-0.40	-0.33	-0.03	0.05
**Glutamic acid (Glu)**	0.19	-0.07	0.37	-0.19	-0.58*	-0.04
**Glycine (Gly)**	-0.10	-0.26	0.23	0.11	-0.47	-0.24
**Alanine (Ala)**	-0.10	0.01	-0.11	0.42	-0.29	-0.16
**Cysteine (Cys)**	-0.11	-0.25	-0.24	-0.05	-0.1	0.15
**Valine (Val)**	-0.50	-0.33	-0.52*	0.55*	0.26	-0.35
**Methionine (Met)**	-0.11	-0.32	0.15	0.14	0.2	-0.19
**Isoleucine (Ile)**	0.31	0.12	0.57*	-0.27	-0.12	0.38
**Leucine (Leu)**	0.04	-0.14	0.45	-0.07	-0.03	0.06
**Tyrosine (Tyr)**	0.35	0.36	0.04	0	-0.2	0.28
**Phenylalanine (Phe)**	-0.07	-0.07	0.02	0.31	0.08	-0.04
**Histidine (His)**	0.23	0.35	-0.16	0.15	-0.02	0.2
**Lysine (Lys)**	0.17	0.33	0.03	-0.06	0.01	0.15
**Ammonia**	-0.30	-0.62*	0.23	-0.09	-0.12	-0.58*
**Arginine (Arg)**	-0.01	0.01	0.22	-0.2	0.22	0.06
**Proline (Pro)**	-0.56*	-0.38	-0.65**	0	-0.06	-0.25

* = significant at *P* < 0.05; ** = significant at *P* < 0.01. Fibre diameter (FD), Prickle factor (PF), medullated fibre (MF), Staple length (SL), Staple strength (SS), Elongation (EL).

## Discussion

### Physical characteristics

Variations in the observed traits compared to literature values in our study may be attributed to differences in geographical representation, sampling methods, and analysis; for instance, Iniguez et al. (2014), who reported no significant differentiation in fiber diameter based on camel sex; in contrast, Annageldiyev et al. (2005) reported lower average fiber diameters of 21.9 μm and 18.8 μm for male and female Arvana dromedary breed, respectively, compared to the averages observed in our research for Sudanese camel-hair fibers. The overall means of fibre diameter shown in the present study (Table 2) exceeded those reported by Rhoades (2007) of Bactrian camels raised in the desert of the USA (18–19 μm), Ansari-Renani et al. (2012) on female dromedaries in Iran (18.6–20.7 μm) and Sharma and Pant (2013) on Sudanese camel-hair (23.49 μm). On the other hand, Salehi (2010) show that on dromedary camel (16.8–39.2 μm) and Helal (2015) on Maghrabi camel-hair (37.9 μm). The obtained results (Table 2) showed that the Sudanese camel’s CV% of fibre diameter that reflected variability between individual samples was 16.67%, which expresses heterogeneity for fibre diameter trait. This is in harmony with the findings of Iniguez et al. (2014), who found that the overall CV% of fibre diameter was 15%.

The overall mean of medullated fibre percentage for Sudanese camel-hair in the present study is lower than that reported (63 to 73%) by Singh and Patni (2004) whose samples contain a mixture of guard hair and fine fibres.

Our results showed insignificant differences among the studied groups, camels of sex, for the staple length of camel- hair fibres. It contrasts with the results of Sahaniet al. (2006), who reported significant effects for sex on staple length of camel’s hair. Meanwhile, Iniguez et al. (2014) reported a non-significant effect for staple length due to sex, which agrees with the results of the present study. In the study of Sharma and Pant (2013) on Sudanese camel-hair staple length was 7.08 cm, which is lower than that presented in the present study. On the other hand, Helal (2015) found that staple length of Maghrabi camels-hair was 14.45 cm longer than recorded in this work.

Staple strength plays a significant role in fibre breakage during mechanical processing, yarn, and fabric strength, and fabric manufacturing and the elongation property of fibres indicated that fibres are more suitable to be used for textile purposes (Harizi et al., 2007). The overall mean of staple strength and elongation for Sudanese camel-hair in our study is (43.14 N/Ktex and 13.05% respectively) which matches with that recorded by Helal (2015) on Magrabi camel-hair (42.83 N/Ktex).

### Chemical composition of camels’ fibers

Animal fibres are composed mainly of keratin protein that comprises eighteen amino acids (McGregor et al., 2018). Their chemical composition, on a mass (weight) basis, is typically 16-17% nitrogen, 3.2-3.7% sulfur, 0.38-0.42% ash (which includes 0.09- 0.12% calcium, 0.017-0.023% phosphorus and some small amounts of sodium), 27% oxygen, 47% carbon and 6% hydrogen (McGregor et al., 2018).

### Fibre minerals content

The minerals could significantly affect fibre and staple quality and, subsequently the yarn properties (Helal 2015). Despite the fact that fibre includes considerable amounts of calcium, potassium, sodium, zinc, copper, manganese, iron, and selenium, only copper, zinc, iodine, and perhaps selenium affect follicle function and fibre growth, as reported by Lee and Grace (1988). Furthermore, Sahoo and Soren (2011) stated that the macro-mineral sulfur acting a significant role in wool production, while the micro-mineral copper plays a very important role in maintaining wool quality. Sulfur plays a very important role in all keratin fibers characteristics as responsible for disulfide bond (Helal 2015). Our results showed that coarse fibers have low contained of sulfur. This might be related to the presence of medulla. The same result was found by Lee and Williams (1996) who stated that fine wool had higher levels of sulfur in wool than strong and medium wool.

In the meantime, camel hair is a good indicator of pollution because of the high exposure of their feed to soil contamination (Mora, et al. 2000). Rashed and Soltan, (2002) found that lead was higher in camel hair than in wool and goat hair. The present results showed that camel-hair of females had high amount of boron, cadmium, iron, manganese and nickel whereas camel hair fibers of males had the high amount of zinc and lead. This finding supported by Helal (2015) who found that fine camel-hair had high amount of cadmium, cobalt, iron, manganese and nickel whereas coarse camel hair fibers had the high amount of molybdenum, zinc and lead.

Faraz et al. (2021) recorded significant differences between males and females for some macro (magnesium and calcium) and trace minerals (zinc, copper, manganese, and iron) of Marecha hair-camels fibre, since their values are more significant in males than a female that is in contrary with our findings in Table (3). The amount of calcium in female and male hair samples of Marecha hair-camels were 486.0 and 521.6 mg/dL, respectively (Faraz et al., 2021), while it ranged from 434.4 to 719.7 mg/dL on dromedary camel calves’ hair (Bhakatet al., 2009).

The overall mean values for calcium, sulfur, potassium, iron, sodium, and aluminum elements in the present study were comparable to those reported by Helal (2015) for Maghrabi camels (4533, 2936, 2247, 532.4, 588, and 533.1 ppm, respectively). In contrast, Faraz et al. (2021) reported different values for Marecha hair-camels (239.9 and 322.2 mg/dL).The overall mean values of silicon, zinc, strontium, chromite, manganese, copper, and nickel in the present study closely resembled those reported by Helal (2015) for the same elements, specifically 134.3, 120.7, 39.95, 36.79, 25.25, 23.49, and 18.46 ppm, respectively. However, our zinc and copper values were lower than those reported by Faraz et al. (2021) (43.8–65.3 and 4.3–7.1 mg/dL, respectively) and Bhakat et al. (2009) (54.76–66.04 and 4.3–7.36 mg/dL, respectively). Additionally, our manganese value was lower than those reported by Faraz et al. (2021) and Bhakat et al. (2009) (25.4 to 46.5 and 20.6 to 45.8 mg/dL, for the two authors, respectively).

### Fibre amino acids and ammonia contents

Cystine plays a curcial role affecting the fibrous protein, fibre content and yarn properties (Stapleton, 1992). In the present study coarse camel hair fibre contains significantly higher amount of glutamic acid, methionine and isoleucine, whereas fine fibers contain higher proline. In the meantime, the amount of glutamic acid, arginine, serine and cysteine in present study were near to Helal (2015) who reported that the amount of the same amino acids were 181.2, 111.9, 108.2, and 105.9 mg/gm, respectively) as reported by Helal (2015) that amino acids content in camels’ fibres were higher in the coarse fibres than the fine ones.

### Association between physical characteristics and chemical composition of camel-hair fibres

Regarding the correlation between the physical characteristics of camel-hair fibers, it has generally been assumed that the prickle factor is linearly correlated with fiber diameter (Whiteley and Thompson, 1985, and Helal et al., 2019). In line with our study, Helal et al. (2019) reported similar findings for Barki sheep wool (*r* = 0.90 and 0.54 for fiber diameter with both prickle factor and medullated fiber, respectively). Similarly, a higher level of coarse fiber content was associated with a more prickly fabric (Dolling et al., 1992). Additionally, Abdel-Moneim et al. (2000) reported a significant and positive correlation coefficient between fiber diameter and medullated fiber. Boominaton et al. (1983) Patkowska et al. (1988) found that sulfur concentration in wool correlated negatively with fiber diameter. Also, Ritchie et al. (1999) clarified that addition of sulfur to the diet reduced fiber diameter of wool growth. Similarly, Helal et al. (2019) reported that the correlation coefficient between fiber diameter and sulfur content in Magrabi camel hair − 0.64.

## Conclusion

The results of our study shed a light on the analysis of the physical and chemical properties of Sudanese camel fibers based on the animals’ sex under local desert harsh condition and the correlation between these traits results indicated a significance effect of sex on some parameters such as potassium content and glutamic acid. A significant positive correlation was detected also between some parameters where Fibre diameter had positive correlations with prickle factor and medullated fibres. Zinc content in camel fibres was positively correlated with fibre diameter and medullated fibres. Through this relationship, there is the possibility of improving the fibre quality and the subsequent fibre manufacturing.

## Data Availability

The data and materials of this study will be available upon request from the correspondence author.
